# Parental Cancer History and Its Association With Minor Children’s Unmet Food, Housing, and Transportation Economic Needs

**DOI:** 10.1001/jamanetworkopen.2023.19359

**Published:** 2023-06-22

**Authors:** Zhiyuan Zheng, Xuesong Han, Jingxuan Zhao, Qinjin Fan, K. Robin Yabroff

**Affiliations:** 1 Surveillance and Health Equity Science, American Cancer Society, Atlanta, Georgia

## Abstract

**Importance:**

A cancer diagnosis is associated with substantial economic burden for patients and their families. Young adult cancer survivors with dependent children may be particularly vulnerable to financial hardship.

**Objective:**

To examine associations of parental cancer with their children’s unmet economic needs.

**Design, Setting, and Participants:**

This cross-sectional study used data from the nationally representative 2013 to 2018 US National Health Interview Survey. Children aged 5 to 17 years living in families with and without parental cancer history were queried about recent 1-year experiences. Statistical analyses were conducted from January 2022 to April 2023.

**Exposure:**

Parental cancer history.

**Main Outcomes and Measures:**

The main outcomes were children’s unmet economic needs, including family-level food insecurity, parent’s financial worry about paying for monthly bills and housing costs, and delayed child medical care owing to lack of transportation. Multivariable logistic regressions adjusted for (1) child’s characteristics (ie, age group, sex, and race and ethnicity), (2) parent’s characteristics (ie, age group, sex, health insurance coverage, comorbid conditions, and obesity status), and (3) family’s characteristics (ie, family structure [married or cohabiting parents vs single parent families], highest educational attainment in the family, and family income). Additional analyses focused on children with a parental cancer history to identify potentially modifiable characteristics associated with unmet economic needs.

**Results:**

In this cross-sectional study of 22 941 children with (812 children; weighted number, 860 488 children) and without (22 129 children; weighted number, 24 545 463 children) a parental cancer history, the majority of children were aged 5 to 11 years (12 022 children [52.4%]), male (11 920 children [52.0%]), and non-Hispanic White (11 863 children [51.7%]). In adjusted analyses, parental cancer history was associated with more severe family-level food insecurity, including worrying about food running out (odds ratio [OR], 1.97; 95% CI, 1.56-2.49; *P* < .001), food not lasting (OR, 2.01; 95% CI, 1.56-2.58; *P* < .001), and inability to afford balanced meals (OR, 1.38; 95% CI, 1.06-1.79; *P* = .02). Moreover, parental cancer history was associated with parent’s worry about paying monthly bills (OR, 1.41; 95% CI, 1.15-1.74; *P* = .001) and housing-related costs (OR, 1.31; 95% CI, 1.07-1.60; *P* = .009) and delays in child medical care because of lack of transportation (OR, 2.31; 95% CI, 1.49-3.59; *P* < .001). Among children with parental cancer history, female children, non-Hispanic Black children, children whose parents had multiple comorbidities, and children living in low-income families were especially vulnerable to unmet economic needs.

**Conclusions and Relevance:**

Parental cancer is associated with greater likelihood of food insecurity, unaffordability of housing and other necessities, and transportation barriers to medical care for minor children. Strategies to identify such children and address their needs are warranted.

## Key Points

**Question:**

What are the associations of parental cancer and minor children’s unmet economic needs related to inadequate food, housing, and transportation in the US?

**Findings:**

In this cross-sectional study of 22 941 children aged 5 to 17 years, parental cancer was associated with food insecurity, unaffordability of housing and other monthly bills, and transportation barriers to medical care. Children with a parental cancer history and living in low-income families were especially vulnerable to unmet economic needs.

**Meaning:**

These findings suggest that strategies to identify children with a parental cancer history and address their unmet economic needs are warranted.

## Teaser

This cross-sectional study examines the associations of parental cancer with their children’s unmet economic needs, including food insecurity, unaffordability of housing and other monthly bills, and transportation barriers to medical care for the children.

## Introduction

Cancer survivors often experience high out-of-pocket medical expenses and financial hardship, with the highest prevalence of hardship among young adult cancer survivors.^1,2,3^ Child-rearing responsibilities may be a contributing factor to financial hardship among young adult cancer survivors.^4^ Previous research^5,6^ found that parental cancer history was associated with worse health among children, including psychosocial and behavioral problems, poor mental health, and school absenteeism due to health problems. Parents with a cancer history may be forced to make trade-offs in allocating limited household financial resources between their own medical needs and basic economic needs of their family.

Raising a child has become more costly in recent years, especially with increasing prices of food, housing, child care, and education.^7^ Patient out-of-pocket costs for cancer treatments have also increased.^8^ Families with a parental cancer history may experience financial hardship for prolonged periods and struggle to maintain their standard of living, even years after diagnosis.^9^ However, little is known about the associations of parental cancer with their children’s basic economic needs related to food, housing, and transportation.

In this cross-sectional study, we used nationally representative data to examine the financial implications of parental cancer on children’s daily living necessities. With a growing interest in adding nonclinical services in medical insurance to address health-related social needs,^10^ this study can help inform policies to screen for and address these children’s unmet economic needs.

## Methods

The 2013 to 2018 National Health Interview Survey (NHIS) was used to identify children aged 5 to 17 years with and without a parental cancer history. The NHIS is a cross-sectional, nationally representative household survey conducted annually in the civilian, noninstitutionalized population in the US, with the annual sample child response rate ranging between 63.4% and 70.7% during these years.^11^ Within each household, a sample child can be linked to a sample adult if the adult was identified as a parent for the child and lived in the same household. Parents with a history of cancer were defined as those who reported receiving a diagnosis of cancer or any malignant neoplasm (termed *malignancy* in the NHIS) by a physician or other health professional.

 This study does not require institutional review board review or informed consent because we used deidentified publicly available data, in accordance with 45 CFR §46. Because NHIS is cross-sectional annual household survey data, there was no loss to follow-up of the survey respondents. We followed the Strengthening the Reporting of Observational Studies in Epidemiology (STROBE) reporting guidelines.^12^

### Statistical Analysis

Children’s unmet economic needs for daily living necessities included family-level food insecurity (ie, worry about food running out, food not lasting, and inability to afford balanced meals), parent’s worry about paying monthly bills and housing-related costs, and delaying child medical care owing to lack of transportation (see the eTable in Supplement 1 for exact wording of questions and response options). We examined both unadjusted and adjusted associations of parental cancer and children’s unmet economic needs. In multivariable logistic regressions, we adjusted for (1) child’s age, sex, and race and ethnicity; (2) parent’s age, sex, health insurance coverage, comorbid conditions, and obesity status; and (3) family’s structure (married or cohabiting parents vs single parent families), highest educational attainment in the family, and family income as a percentage of the federal poverty level (FPL). Both parent’s and child’s race and ethnicity were included in our study. Parent’s race and ethnicity information was reported by the sample parent herself or himself, and the child’s race and ethnicity information was reported by an adult in the family who had the most knowledge about the sample child’s health and may not be the sample parent interviewed. All categories were classified by the NHIS. We used reported race and ethnicity information and grouped children and parents into 4 groups: Hispanic, non-Hispanic Black, non-Hispanic White, and Asian and others (ie, Hispanic Black, Native American and Alaska Native, multiple race, and unknown). We treated race and ethnicity data as a sociodemographic factor because previous literature has shown that it is associated with health disparities in the US.^13^ Survey year and geographic region were also included in the multivariable models.

Two summary measures were created for any family-level food insecurity and any parent’s financial worry about paying monthly bill and housing-related costs, respectively. Additional adjusted logistic regressions were conducted among children with a parental cancer history to examine the associations of child, parent, and family characteristics with the 2 summary measures. In addition to all the child, parent, and family characteristics, time since diagnosis of the parent with a history of cancer was added to the multivariable regressions.

χ^2^ tests were used to compare distributions of child, parent, and family characteristics between children with and without a parental cancer history. For children’s unmet economic needs, all estimates were obtained through unadjusted and adjusted logistic regressions. Statistical analyses were conducted using SAS statistical software version 9.4 (SAS Institute) and Stata statistical software version 16.1 (StataCorp). All comparisons were 2-sided with significance defined as *P* < .05. Statistical analyses were conducted from January 2022 to April 2023.

## Results

In this cross-sectional study of nationally representative children aged 5 to 17 years, the final sample consisted of 22 941 children, including 812 with (weighted number, 860 488) and 22 129 without (weighted number, 24 545 463) parental cancer history (Table 1). The majority of children were aged 5 to 11 years (12 022 children [52.4%]), male (11 920 children [52.0%]), and non-Hispanic White (11 863 children [51.7%]). Compared with children without a parental cancer history (Table 1), children with a parental cancer history were older and were more likely to be non-Hispanic White and to live with a single parent. Compared with parents without a cancer history, parents with a cancer history were older, more likely to be female and non-Hispanic White, to have public insurance, and to have more comorbid conditions.

**Table 1.** Characteristics of Minor Children Living in Families With and Without a Parental Cancer History, National Health Interview Survey 2013-2018

Characteristics	Children, No. (weighted %)		*P *value^a^
	With parental cancer history (n = 812; weighted n = 860 488 [3.4%])	Without parental cancer history (n = 22 129; weighted n = 24 545 463 [96.6%])	
Child characteristics			
Age of minor child, y			
5-11	335 (45.7)	11 687 (55.8)	<.001
12-14	201 (22.7)	5069 (22.7)	
15-17	276 (31.7)	5373 (21.5)	
Sex of minor child			
Male	429 (50.2)	11 491 (51.1)	.68
Female	383 (49.8)	10 638 (48.9)	
Race and ethnicity of minor child			
Asian and others^b^	52 (5.7)	1782 (6.9)	<.001
Hispanic	142 (17.3)	5607 (23.8)	
Non-Hispanic Black	91 (11.7)	3404 (15.9)	
Non-Hispanic White	527 (65.4)	11 336 (53.4)	
Parent characteristics			
Age of parent, y			
18-39	290 (42.2)	10 202 (49.8)	<.001
40-64	502 (55.6)	11 820 (49.9)	
≥65	20 (2.2)	107 (0.4)	
Sex of parent			
Male	148 (16.8)	6394 (29.1)	<.001
Female	664 (83.2)	15 735 (70.9)	
Race and ethnicity of parent			
Asian and others^b^	37 (4.7)	1618 (6.4)	<.001
Hispanic	115 (13.5)	5182 (21.9)	
Non-Hispanic Black	72 (8.8)	3251 (15.2)	
Non-Hispanic White	588 (73.0)	12 078 (56.6)	
Health insurance of parent			
Aged 18-64 y, any private	452 (52.9)	13 572 (61.3)	<.001
Aged 18-64 y, public only	243 (32.0)	4797 (22.4)	
Aged 18-64 y, uninsured or missing	97 (12.9)	3653 (15.9)	
Aged ≥65 y, Medicare with or without other insurance	20 (2.2)	107 (0.4)	
No. of comorbid conditions of parent^c^			
0	429 (54.5)	15 119 (70.1)	<.001
1	243 (31.1)	5566 (24.3)	
2	102 (10.5)	1176 (4.6)	
≥3	38 (3.9)	268 (1.0)	
Body mass index of parent^d^			
18.5 to <25	273 (38.1)	7361 (34.0)	.05
<18.5	14 (2.2)	257 (1.0)	
25 to <30	233 (27.2)	7073 (32.0)	
≥30	292 (32.4)	7438 (32.9)	
Time since diagnosis of parental cancer			
Recent diagnosis (<2 y)	157 (18.8)	NA	NA
Previous diagnosis (≥2 y)	655 (81.2)	NA	
Family characteristics			
Family structure			
Married or cohabiting parents	505 (64.7)	14 853 (69.8)	.02
Single parent	307 (35.3)	7276 (30.2)	
Highest education in the family			
Less than high school	99 (11.7)	2878 (13.1)	.09
High school graduate	103 (12.0)	3420 (14.8)	
Some college or higher	610 (76.2)	15 831 (72.1)	
Family income, % of federal poverty level			
<200	358 (46.1)	9215 (42.6)	.12
≥200 to <400	218 (25.6)	5793 (25.6)	
≥400	214 (25.0)	6025 (26.6)	
Missing	22 (3.3)	1096 (5.2)	
Other characteristics			
Survey year			
2013	133 (16.7)	4310 (16.8)	.20
2014	151 (15.2)	4479 (16.9)	
2015	139 (14.9)	4092 (16.9)	
2016	163 (20.0)	3627 (16.4)	
2017	130 (18.1)	2804 (16.1)	
2018	96 (15.0)	2817 (16.9)	
Region			
Northeast	114 (12.4)	3428 (16.1)	.05
Midwest	202 (28.6)	4676 (23.1)	
South	296 (37.1)	8030 (37.9)	
West	200 (21.9)	5995 (22.9)	

Abbreviation: NA, not applicable.

^a^ The reference group was children without parental cancer, and *P* values were obtained by comparing weighted percentages. Parents with only nonmelanoma skin cancer or skin cancer of unknown type were excluded. All statistical tests were 2 sided, and all *P* values were calculated using Pearson χ^2^ test statistic. All numbers were raw numbers, and all percentages were weighted column percentages.

^b^ All categories were classified by the National Health Interview Survey. Others included those who reported multiple races, Hispanic Black, Native American and Alaska Native, or unknown.

^c^ The number of chronic conditions was defined as the sum of the following conditions: that a respondent was ever told by a doctor or other health professional that they had arthritis, asthma, cancer, diabetes, emphysema, heart disease (angina, coronary heart disease, heart attack, or another heart condition or disease), high cholesterol, hypertension, stroke, and cancer.

^d^ Body mass index is calculated as weight in kilograms divided by height in meters squared.

In adjusted analyses, parental cancer history was associated with more severe family-level food insecurity, including worry about food running out (30.1% [95% CI, 26.5%-33.7%] vs 20.1% [95% CI, 19.5%-20.7%]; odds ratio [OR], 1.97 [95% CI, 1.56-2.49]; *P* < .001), food not lasting (26.0% [95% CI, 22.3%-29.7%] vs 16.7% [95% CI, 16.1%-17.3%]; OR, 2.01 [95% CI, 1.56-2.58]; *P* < .001), and inability to afford balanced meals (16.9% [95% CI, 13.8%-20.0%] vs 13.3% [95% CI, 12.8%-13.8%]; OR, 1.38 [95% CI, 1.06-1.79]; *P* = .02) (Table 2). Moreover, parents with a cancer history were more likely than those without a cancer history to report worries about paying monthly bills (44.8% [95% CI, 40.6%-48.9%] vs 37.9% [95% CI, 37.1%-38.7%]; OR, 1.41 [95% CI, 1.15-1.74]; *P* = .001) and housing-related costs (35.7% [95% CI, 31.9%-39.5%] vs 30.7% [95% CI, 30.0%-31.5%]; OR, 1.31 [95% CI, 1.07-1.60]; *P* = .009) and to experience delays in child medical care because of lack of transportation (3.6% [95% CI, 2.2%-4.9%] vs 1.6% [95% CI, 1.4%-1.9%]; OR, 2.31 [95% CI, 1.49-3.59]; *P* < .001) (Table 2). Unadjusted results were consistent with the adjusted results (Table 2).

**Table 2.** Associations of Parental Cancer History With Food Insecurity, Unaffordability of Monthly Bills and Housing, and Lack of Transportation for Child Medical Care, National Health Interview Survey 2013-2018

Variable	Children, % (95% CI)		Children with parental cancer vs children without parental cancer^a^	
	With parental cancer	Without parental cancer	OR (95% CI)	*P* value
Unadjusted unmet economic needs among children aged 5-17 y^b^				
Often or sometimes true that worry about food running out	34.0 (29.4-38.6)	20.0 (19.3-20.8)	2.06 (1.67-2.53)	<.001
Often or sometimes true that food not lasting	29.3 (24.7-33.9)	16.6 (15.9-17.3)	2.08 (1.66-2.61)	<.001
Often or sometimes true that unable to afford balanced meals	19.9 (16.2-23.6)	13.2 (12.6-13.8)	1.63 (1.29-2.06)	<.001
Very or moderately worried about monthly bills	47.5 (42.8-52.2)	37.8 (36.9-38.8)	1.48 (1.22-1.80)	<.001
Very or moderately worried about housing costs	37.3 (32.7-41.8)	30.7 (29.8-31.5)	1.34 (1.10-1.63)	.003
Delayed child medical care because of lack of transportation	4.7 (2.8-6.7)	1.6 (1.4-1.9)	3.02 (1.92-4.78)	.002
Adjusted unmet economic needs among children aged 5-17 y^b^				
Often or sometimes true that worry about food running out	30.1 (26.5-33.7)	20.1 (19.5-20.7)	1.97 (1.56-2.49)	<.001
Often or sometimes true that food not lasting	26.0 (22.3-29.7)	16.7 (16.1-17.3)	2.01 (1.56-2.58)	<.001
Often or sometimes true that unable to afford balanced meals	16.9 (13.8-20.0)	13.3 (12.8-13.8)	1.38 (1.06-1.79)	.02
Very or moderately worried about monthly bills	44.8 (40.6-48.9)	37.9 (37.1-38.7)	1.41 (1.15-1.74)	.001
Very or moderately worried about housing costs	35.7 (31.9-39.5)	30.7 (30.0-31.5)	1.31 (1.07-1.60)	.009
Delayed child medical care because of lack of transportation	3.6 (2.2-4.9)	1.6 (1.4-1.9)	2.31 (1.49-3.59)	<.001

Abbreviation: OR, odds ratio.

^a^ The reference group was minor children without a parental cancer history, and the *P* values were obtained from logistic regressions. All statistical tests were 2-sided, and all *P* values were generated from multivariable logistic regression analyses. All regressions were adjusted for child’s age, sex, and race or ethnicity; parent’s age, sex, health insurance coverage, number of comorbid conditions, and obesity status; family’s structure (married or cohabiting parents vs single parent families); highest educational attainment in the family; and family income as a percentage of the federal poverty level. US geographic region and survey year were also included in adjusted analyses.

^b^ See the eTable in Supplement 1 for exact wording of questions and response options.

Among children with a parental cancer history, characteristics that were associated with food insecurity included female vs male sex (OR, 1.61; 95% CI, 1.03-2.51, *P* = .04), non-Hispanic Black vs non-Hispanic White race (OR, 2.28; 95% CI, 1.04-4.99; *P* = .04), the parent being covered by public insurance vs private insurance (OR, 1.89, 95% CI, 1.04-3.43; *P* = .04), the parent having multiple comorbid conditions vs no comorbid condition (OR, 2.83; 95% CI, 1.39-5.73; *P* = .004), and low family income (<200% FPL vs ≥400% FPL: OR, 20.88; 95% CI, 8.70-50.11; *P* < .001; ≥200% and <400% FPL vs ≥400% FPL: OR, 5.32; 95% CI, 2.31-12.27; *P* < .001) (Table 3). Among children with a parental cancer history, characteristics that were associated with parent’s financial worry about monthly bills and housing costs included non-Hispanic Black vs non-Hispanic White race (OR, 2.11; 95% CI, 1.05-4.27; *P* = .04), the parent having multiple comorbid conditions vs no comorbid condition (OR, 1.82; 95% CI, 1.03-3.21; *P* = .04), the parent being underweight (OR, 7.87; 95% CI, 1.57-39.41; *P* = .01), and low family income (<200% FPL vs ≥400% FPL: OR, 4.24; 95% CI, 2.17-8.29; *P* < .001; ≥200% and <400% FPL vs ≥400% FPL: OR, 1.79; 95% CI, 1.04-3.08, *P* < .001) (Table 3).

**Table 3.** Adjusted Results of Characteristics Associated With Unmet Economic Needs Among Children Living in Families With a Parental Cancer History, National Health Interview Survey 2013-2018

Children with a parental cancer history (n = 812; weighted n = 860 488)	Any family-level food insecurity^a^^,^^b^		Any parent’s financial worry^a^^,^^c^	
	OR (95% CI)	*P* value^d^	OR (95% CI)	*P* value^d^
Age of minor child, y				
5-11	1 [Reference]	NA	1 [Reference]	NA
12-14	1.19 (0.67-2.14)	.55	1.50 (0.92-2.46)	.11
15-17	0.97 (0.55-1.70)	.91	1.11 (0.68-1.82)	.68
Sex of minor child				
Male	1 [Reference]	NA	1 [Reference]	NA
Female	1.61 (1.03-2.51)	.04	1.45 (0.98-2.17)	.07
Race or ethnicity of minor child				
Asian and others^e^	0.61 (0.25-1.51)	.29	0.88 (0.4-1.92)	.75
Hispanic	0.98 (0.54-1.78)	.96	1.22 (0.71-2.07)	.47
Non-Hispanic Black	2.28 (1.04-4.99)	.04	2.11 (1.05-4.27)	.04
Non-Hispanic White	1 [Reference]	NA	1 [Reference]	NA
Age of parent, y				
18-39	1 [Reference]	NA	1 [Reference]	NA
40-64	1.13 (0.67-1.92)	.65	0.87 (0.52-1.45)	.59
≥65	1.49 (0.45-4.92)	.52	0.4 (0.11-1.49)	.17
Sex of parent				
Male	1 [Reference]	NA	1 [Reference]	NA
Female	1.92 (0.96-3.81)	.06	1.17 (0.67-2.04)	0.59
Health insurance of parent				
Aged 18-64 y, any private	1 [Reference]	NA	1 [Reference]	NA
Aged 18-64 y, public only	1.89 (1.04-3.43)	.04	1.49 (0.85-2.63)	.17
Aged 18-64 y, uninsured or missing	1.97 (0.91-4.24)	.08	1.90 (0.99-3.65)	.05
Aged ≥65 y, Medicare with or without other insurance	NA	NA	NA	NA
No. of comorbid conditions of parent				
0	1 [Reference]	NA	1 [Reference]	NA
1	1.36 (0.80-2.30)	.25	1.60 (1.00-2.55)	.05
≥2	2.83 (1.39-5.73)	.004	1.82 (1.03-3.21)	.04
Body mass index of parent^f^				
18.5 to <25	1 [Reference]	NA	1 [Reference]	NA
<18.5	5.10 (1.01-25.63)	.05	7.87 (1.57-39.41)	.01
25 to <30	0.84 (0.48-1.46)	.53	1.41 (0.87-2.28)	.17
≥30	0.65 (0.38-1.13)	.13	1.00 (0.60-1.65)	>.99
Time since diagnosis of parental cancer				
Recent diagnosis (<2 y)	1 [Reference]	NA	1 [Reference]	NA
Previous diagnosis (≥2 y)	1.14 (0.64-2.02)	.66	0.72 (0.43-1.20)	.21
Family structure				
Married or cohabiting parents	1 [Reference]	NA	1 [Reference]	NA
Single parent	1.26 (0.76-2.09)	.37	1.26 (0.82-1.94)	.30
Highest education in the family				
Some college or higher	1 [Reference]	NA	1 [Reference]	NA
High school graduate	1.04 (0.51-2.12)	.92	0.65 (0.33-1.27)	.21
Less than high school	1.47 (0.78-2.76)	.23	0.75 (0.39-1.44)	.39
Family income, % of federal poverty level				
≥400	1 [Reference]	NA	1 [Reference]	NA
≥200 to <400	5.32 (2.31-12.27)	<.001	1.79 (1.04-3.08)	.03
<200	20.88 (8.70-50.11)	<.001	4.24 (2.17-8.29)	<.001
Missing	3.07 (0.56-16.75)	.20	1.78 (0.6-5.32)	.30

Abbreviations: NA, not applicable; OR, odds ratio.

^a^ See the eTable in Supplement 1 for exact wording of questions and response options.

^b^ The food insecurity summary measure included any family-level food insecurity (ie, worry about food running out, food not lasting, and unable to afford balanced meals).

^c^ The financial worry summary measure included 2 parent’s financial worry measures (ie, worry about paying monthly bills or housing-related costs).

^d^ All statistical tests were 2-sided, and all *P* values were generated from multivariable logistic regression analyses. All regressions were adjusted for child’s age, sex, and race or ethnicity; parent’s age, sex, health insurance coverage, number of comorbid conditions, obesity status, and time since cancer diagnosis; family’s structure (married or cohabiting parents vs single parent families); highest educational attainment in the family; and family income as a percentage of the federal poverty level. US geographic region and survey year were also included in adjusted analyses.

^e^ All categories were classified by the National Health Interview Survey. Others included those who reported multiple races, Hispanic Black, Native American and Alaska Native, or unknown.

^f^ Body mass index is calculated as weight in kilograms divided by height in meters squared.

## Discussion

In this nationally representative cross-sectional study, we found that children with a parental cancer history were more likely to experience food insecurity, unaffordability of housing and other living necessities, and transportation barriers to medical care compared with children without a parental cancer history. Moreover, non-Hispanic Black children, children whose parents had poor health, and children living in low-income families were more likely to experience food insecurity and parent’s financial worry about paying monthly bills and housing costs. Our findings add to the accumulating evidence that a cancer diagnosis can adversely affect physical and mental health and financial burden for other family members, including spouses and other informal caregivers.^14,15,16,17^ Our findings also suggest that efforts to identify children with a parental cancer history and screen for any unmet economic needs and connect them with relevant services are warranted.

The vulnerability of young adult cancer survivors to medical financial hardship is well documented.^2,3,18^ Their coping strategies for high out-of-pocket medical costs include delaying or forgoing medical care and prescription medication to save money.^1^ This study expands the evidence base by examining the financial implications of parental cancer on children’s daily living necessities. Younger adult cancer survivors with children may have lower wealth accumulation and higher debt obligations (eg, paying off home mortgages and student loans) than their older counterparts,^9^ which may force them to make trade-offs to provide sufficient housing, food, and child care for their children. A recent estimate from the US Department of Agriculture for annual costs of raising a child ranges from $15 438 to $17 375 in 2022, which represents a 23.3% increase compared with the year 2015.^19^ Another estimate shows that a middle income family is expect to spend, on average, $310 605 to raise a child born in 2015 until age 17 years.^20^ Because of the increasing costs of both cancer treatments and raising children, all family members of the young adult parents with a history of cancer may be particularly vulnerable to unmet economic needs.

Previous research^14^ on parental cancer history found worse physical and mental health outcomes among minor children. However, the potential pathways for these associations were unclear. Our findings provide important perspectives for understanding the association of parental cancer with poor health among minor children. Food insecurity has been consistently associated with poor health among children.^21^ Compared with food-secure children, food-insecure children were more likely to have asthma, iron deficiency anemia, and tooth decay and to experience poor mental health, such as depression, anxiety, and suicide ideation.^21^ Poor living conditions, such as lead exposures or lack of adequate heating and cooling at home, may also cause illnesses among children.^22^ Combined with the finding of transportation barriers for child medical care, our study provides evidence that can inform interventions to identify and mitigate the negative associations of parental cancer on children’s health, as well as their basic economic needs for daily living.

The Centers for Medicare & Medicaid Services recently allowed states to apply for waivers to address health-related social needs in their Medicaid programs, including services related to food and housing, education, and employment,^10,23^ which allows individual states to start experimental or pilot programs that provide nonmedical benefits to low-income populations under their Medicaid and Children’s Health Insurance Program.^24,25^ For example, New Jersey, Arkansas, Massachusetts, and Oregon can now use Medicaid funds to assist beneficiaries with rent support or health-related home improvements, such as mold removal.^26^ Moreover, most of these states also developed food-as-medicine programs, which can also pay for nutritious food, including fruits and vegetables.^26^ Our results showed that parents with a history of cancer who are economically disadvantaged or have multiple comorbid conditions face high risks of food insecurity and housing instability, which inevitably affect their minor children and other family members. Therefore, children with a parental cancer history and living in states that applied and were approved for waivers may benefit from these initiatives. However, more than half of the states have not applied for these waivers to address health-related social needs.^23^ Evaluation of the effectiveness of these Medicaid pilot programs on addressing health-related social needs in children with a parental cancer history is warranted.

### Limitations

This study has limitations. Measures of food insecurity and financial worry were self-reported by the sample adult. Lack of transportation for child medical care was obtained from an adult respondent in the family who is knowledgeable about the child’s health, who may not be the sample adult. Moreover, we do not have detailed cancer-related information, such as stage of diagnosis and type of treatments received for the parents’ cancer.

## Conclusions

In this cross-sectional study, parental cancer was adversely associated with children’s daily living necessities, including food insecurity, housing unaffordability, and transportation barriers to medical care. Socioeconomically disadvantaged children with a parental cancer history are especially vulnerable. Efforts to identify children with a parental cancer history and develop strategies to mitigate their unmet economic needs are warranted.

## Notes

Supplement 1. **eTable.** National Health Interview Survey Questions and Response Options

Supplement 2.
Data Sharing Statement
