# Supplementary material for: Parental Cancer History and Its Association With Minor Children’s Unmet Food, Housing, and Transportation Economic Needs
Source: JAMA Netw Open. 2023 Jun 22;6(6):e2319359. doi: 10.1001/jamanetworkopen.2023.19359 (PMC10288335; doi:10.1001/jamanetworkopen.2023.19359)
Supplement: Supplement 1. — eTable. National Health Interview Survey Questions and Response Options [file jamanetwopen-e2319359-s001.pdf]

Supplemental Online Content

Zheng Z, Han X, Zhao J, Fan Q, Yabroff KR. Parental cancer history and its association with minor children's unmet food, housing, and transportation economic needs. *JAMA Netw Open*. 2023;6(6):e2319359. doi:10.1001/jamanetworkopen.2023.19359

eTable. National Health Interview Survey Questions and Response Options

This supplemental material has been provided by the authors to give readers additional information about their work.

© 2023 Zheng Z et al. *JAMA Network Open.*

eTable. National Health Interview Survey Questions and Response Options

**Worry about food running out**	*"We worried whether our food would run out before /we got money to buy more. Was that often true, sometimes true, or never true for your family in the last 30 days?" Often and sometimes true were categorized as "yes" versus never true as "no".*
**Food not lasting**	*"The food that we bought just didn't last, and we didn't have money to get more. Was that often true, sometimes true, or never true for your family in the last 30 days?" Often and sometimes true were categorized as "yes" versus never true as "no".*
**Unable to afford balanced meals**	*"We couldn't afford to eat balanced meals. Was that often true, sometimes true, or never true for your family in the last 30 days?" Often and sometimes true were categorized as "yes" versus never true as "no".*
**Worry about monthly bills**	*"How worried are you right now about not being able to pay your rent, mortgage, or other housing costs? Are you very worried, moderately worried, not too worried, not worried at all?" Very and moderately worried were categorized as "yes" versus not too worried and not worried at all were categorized as "no".*
**Worry about housing costs**	*"How worried are you right now about not having enough to pay your normal monthly bills? Are you very worried, moderately worried, not too worried, not worried at all?" Very and moderately worried were categorized as "yes" versus not too worried and not worried at all were categorized as "no".*
**Delayed child medical care due to lack of transportation**	*"There are many reasons people delay getting medical care. Have you delayed getting care for the sample child for any of the following reasons in the PAST 12 MONTHS? ... You didn't have transportation? Yes or no".*

© 2023 Zheng Z et al. *JAMA Network Open.*
