# Supplementary material for: Parental Cancer History and Its Association With Minor Children’s Unmet Food, Housing, and Transportation Economic Needs
Source: JAMA Netw Open. 2023 Jun 22;6(6):e2319359. doi: 10.1001/jamanetworkopen.2023.19359 (PMC10288335; doi:10.1001/jamanetworkopen.2023.19359)
Supplement: Supplement 2. — Data Sharing Statement [file jamanetwopen-e2319359-s002.pdf]

Data Sharing Statement

Zheng. Parental Cancer History and Its Association With Minor Children's Unmet Food, Housing, and Transportation Economic Needs. *JAMA Netw Open*. Published June 22, 2023. doi:10.1001/jamanetworkopen.2023.19359

Data
Data available: Yes
Data types: Deidentified participant data
How to access data: The data is publicly available by CDC.
When available: With publication

Supporting Documents
Document types: None

Additional Information
Who can access the data: It is publicly available to everyone.
Types of analyses: NA
Mechanisms of data availability: NA
Any additional restrictions: NA
